# Interleukin-1 gene polymorphisms and toxoplasmic retinochoroiditis

**Published:** 2008-10-12

**Authors:** Cynthia A. Cordeiro, Paula R. Moreira, Germano C. Costa, Walderez O. Dutra, Wesley R. Campos, Fernando Oréfice, Antônio L. Teixeira

**Affiliations:** 1 Uveitis Section, Department of Ophthalmology, School of Medicine, Belo Horizonte, Brazil; 2 Department of Morphology, Institute of Biological Sciences, Belo Horizonte, Brazil; 3 Department of Internal Medicine, School of Medicine, Federal University of Minas Gerais, Belo Horizonte, Brazil

## Abstract

**Purpose:**

It has been proposed that cytokine gene polymorphisms can predispose individuals to disease by enhancing inflammatory processes. Considering the relevance of interleukin-1 (IL-1) in the pathogenesis of toxoplasmic retinochoroiditis (TR), we investigated whether *IL1A* −889 C/T and *IL1B* +3954C/T promoter polymorphisms are associated with TR in humans.

**Methods:**

We performed a cross-sectional study that involved 100 Brazilian TR patients and 100 age- and gender-matched control subjects. Genomic DNA was obtained from oral swabs of all participants and amplified using polymerase chain reaction (PCR) with specific primers flanking the locus −889 of *IL1A* and +3954 of *IL1B*. PCR products were submitted to digestion and analyzed by PAGE to distinguish C and T alleles.

**Results:**

There was no significant difference in the genotype or allele distributions of the *IL1A* −889 C/T and *IL1B* +3954C/T polymorphisms in patients with TR when compared with controls. However, in a subgroup analysis, the frequency of genotype and allele distributions of IL1A −889 C/T differed significantly between TR patients with and without recurrent episodes.

**Conclusion:**

This study suggests that the genotypes related with a high production of IL-1a may be associated with the recurrence of TR.

## Introduction

Toxoplasmic retinochoroiditis (TR) is the most common cause of posterior uveitis in many parts of the world, especially in Brazil [1]. TR results in inflammation and dysfunction of the retina, occasionally leading to loss of vision. The intensity of damage to the retina and choroid depends on the severity of the infection and of the associated inflammatory reaction [1,2].

While several mediators influence the development of inflammatory responses, interleukin-1 (IL-1) is likely to play a major role in these processes [3]. Produced mainly by activated mononuclear cells, IL-1 is responsible for the induction of adhesion molecules on endothelial cells, thereby facilitating the migration of leukocytes from blood vessels into inflamed tissue [4].

There is considerable evidence obtained from experimental data and from studies involving humans to suggest a relevant role for IL-1 in the pathogenesis of TR. For instance, acquired TR patients have been found to present higher levels of IL-1 than asymptomatic subjects [5]. Additionally, human retinal pigmented epithelial cells inoculated with *Toxoplasma gondii* increase their production of IL-1β, IL-6, granulocyte-macrophage colony-stimulating factor (GM-CSF), and intercellular adhesion molecule (ICAM) [6]. Elevated production of these factors may contribute to the local inflammatory response during primary infections or during recurrent episodes of toxoplasma-induced retinochoroiditis [6].

The IL-1 family consists of three homologous proteins: IL-1α and IL-1β, which are pro-inflammatory proteins, and IL-1 receptor antagonist (IL-1ra), a molecule with antiinflammatory properties. These proteins are encoded by the genes *IL1A*, *IL1B,* and *IL1RN*, respectively, which are clustered on chromosome 2q13–21 and are polymorphic in several loci [7,8]. Single nucleotide polymorphisms in the IL-1 locus, their functional consequences, and their association with susceptibility and severity of inflammatory diseases have been discussed in the literature [9,10]. For instance, the severity of periodontal disease has been positively associated with carriage of allele T at position -889 of the IL-1A gene [11].

Several studies have shown the functional relevance of the promoter region of *IL1A* for the regulation of IL-1 expression [10-12]. The C/T single base variation was described in the −889 locus of the *IL1A* promoter, with the allele T being associated with a fourfold increase of IL-1α expression [11]. This suggests a mechanism whereby this genetic polymorphism acts to modulate IL-1alpha protein production and may influence the pathogenesis of inflammatory diseases, including periodontal disease [11]. Additionally, the *IL1A* (−889) CC genotype has been associated with a significantly lower transcriptional activity of the gene and lower plasmatic levels of IL-1α, when compared with the TT genotype [10].

A polymorphism in the +3954 locus of *IL1B* has been associated with an increased production of IL-1β. Homozygous individuals for the T allele produce a fourfold higher amount of IL-1β compared to individuals displaying the CC genotype [13]. This genetic mediated cytokine production may explain why some subjects have a more vigorous inflammatory response than others to the same stimulus [14].

We hypothesized that the presence of polymorphic alleles (T), related to the expression of higher levels of IL-1α and IL-1β, may be associated with the occurrence of TR. Therefore, our aim was to investigate the possible association between *IL1A* (−889) and *IL1B* (+3954) polymorphisms and TR in humans.

## Methods

### Subjects

This study protocol adhered to the tenets of the Declaration of Helsinki and was approved by the local Institutional Review Board. Patients were informed verbally and in writing of the potential benefits and risks of the study, and all patients signed a written consent form.

Between August and November 2006, 100 patients (41 males, 59 females) with diagnosed TR were recruited from the Uveitis Section, Department of Ophthalmology, Federal University of Minas Gerais - Brazil,. All patients underwent a detailed ocular examination, including corrected visual acuity, applanation tonometry for intraocular pressure, slit lamp examination, a fundus examination with 78 D lens and indirect ophthalmoscope. The number and location of retinochoroidal lesions were documented for all patients by fundus drawings or photographs. Active TR was defined by the presence of gray-white focus of retinal necrosis next to a pigmented retinal scar in patients with positive serology for toxoplasmosis. Recurrent disease was defined as a new active focus of retinal necrosis after three months of an active episode [15].

Additionally recruited were 100 healthy donors from the Hemominas Foundation, Belo Horizonte, Brazil. These donors were matched by age and gender and served as controls. All controls had a positive IgG antibody for toxoplasmosis and no history of uveitis. Each control was given an ocular examination to exclude the presence of retinal scars.

### Sample collection and DNA extraction

Epithelial cells were collected from all patients and controls by buccal swab. Each plastic spatula used to harvest the cells was placed immediately in 1500 μl of Krebs buffer, which contained 20% NaCl, 2% KCl, 2% CaCl_2_, 2% H_2_O, MgSO_4_, KH_2_PO_4_, and C_6_H_12_O_6_. DNA extraction was then performed. A pellet of cells was obtained by centrifugation at 200x g for 5 min. The supernatant was removed. Next, 20 μl of silica (Sigma, St. Louis, MO) and 450 μl of lysis buffer, which consisted of 6.0 M guanidinium thiocyanate (GuSCN) 65 mM Tris, pH 6.4, 25 mM EDTA, and 1.5% Triton X-100, were added to the microtubes. Samples were homogenized and 98 incubated for 30 min at 56 °C. Next, samples underwent another centrifugation, and the supernatant was discharged. The pellet obtained (with DNA adsorbed on the silica) was washed twice with 450 μl washing buffer that consisted of 6.0 M GuSCN and 65 mM Tris, pH 6.4. twice with 450 μl of 70% ethanol, once with 450 μl acetone, and then dried at 56 °C for 20 min. Finally, 100 μl of Tris-EDTA (TE) buffer, which contained 10 mM Tris, pH 8.0 and 1 mM EDTA, was added, and the supernatant was incubated at 56 °C for 12 h to release the DNA. After incubation, the solution was homogenized, centrifuged, and the supernatant containing DNA was transferred to a new tube.

### Polymerase chain reaction and restriction endonuclease digestion

The *IL1A* (−889) polymorphism (Gene ID 3552) was assessed after polymerase chain reaction (PCR) amplification and digestion. The sequences of PCR primers used were as follows: 5'-AAG CTT GTT CTA CCA CCT GAA CTA GGC-3' and 5'-TTA CAT ATG AGC CTT CCA TG-3', with an expected PCR product size of 99 bp for the *IL1-α* polymorphism [7]; and 5'-AGG CAA TAG GTT TTG AGG GCC AT-3' and 5'-TCC TCC CTG CTC CGA TTC CG-3', with an expected PCR product size of 194 bp for the *IL1β* polymorphism (Gene ID 3553), as previously described [16]. PCR was performed in a total volume of 50 μl, which contained 10 μl of DNA solution, 20 pmol primers per reaction, and a premix buffer that consisted of 50 mM KCl, 10 mM Tris, pH 8.4, 0.1% Triton X-100, 1.5 mM MgCl_2_, deoxynucleoside triphosphates, and Taq DNA polymerase. The amplification conditions consisted of 94 °C for 3 min, followed by 45 cycles of 94 °C for 30 s, 56 °C for 35 s, and 72 °C for 30 s. The run was terminated by a final elongation at 72 °C for 5 min. For *IL-1A* polymorphism, 5U of NcoI enzyme was used and the digestion products obtained were C (83+16bp) and T (99 bp) alleles. For *IL-1B* polymorphism, 5U of TaqI enzyme was used and the digestion products obtained were C (97+85+12 bp) and T (182+12 bp) alleles. The visualization of the digestion products was done in a 10% acrylamide gel electrophoresis stained with silver nitrate.

### Statistical analysis

The study groups were tested for Hardy–Weinberg equilibrium by comparing the expected with the observed genotype frequencies.

Statistical analyses were performed using the SPSS for Windows (11.0.1; SPSS, Inc., Chicago, IL). Associations with TR were investigated between genotype and allelic frequencies. χ^2^ analysis and calculation of odds ratio (OR) with 95% confidence interval were performed. The level of statistical significance was set at p<0.05.

## Results

We analyzed genotype and allele distributions of the *IL1A*-889 C/T polymorphism in controls. No difference was found between observed and expected distributions of genotypes for control subjects and, therefore, it was considered to be in Hardy–Weinberg equilibrium. There was no significant difference in the genotype (χ^2^=2.58; p=0.28) or allele (χ^2^=1.91; p=0.17) distribution in patients with TR compared with controls (Table 1). A subgroup analysis was performed involving only patients with one or more years of follow-up (n=59; Table 2). The mean follow-up did not differ significantly between patients with (7.4±3.4 years) and without (6.7±8.6 years) previous TR episodes (p=0.10). The frequencies of the genotype (χ^2^=5.71, p=0.03) and of the allele (χ^2^=5.46, p=0.02) distributions differed significantly when comparing TR patients with and without recurrence of the disease. The CT genotype and T allele were associated with the recurrence of TR.

**Table 1:** Distribution of the IL1A (-889) and IL1B (+3954) genotypes and allele between toxoplasmosis retinochoroiditis patients and control subjects

**Polymorphism**	**TR** **n**	**Control** **n**	**χ^2^**	**p**
IL1A				
Genotype				
CC	48	42	2.58	0.28
CT	44	43		
TT	8	15		
Allele				
C	140	127	1.91	0.17
T	60	73		
IL1B				
Genotype				
CC	49	61	3.12	0.21
CT	46	34		
TT	5	5		
Allele				
C	144	156	1.92	0.17
T	56	44		

Distribution of the IL1A (-889) and IL1B (+3954) genotypes and allele between toxoplasmosis retinochoroiditis (TR) patients (n=100) and control subjects (n=100).

**Table 2:** Distribution of the IL1A (-889) and IL1B (+3954) genotypes and allele in toxoplasmosis retinochoroiditis patients with and without recurrent episodes

Recurrence	Yes	No		
Polymorphism	n	n	χ^2^	p
IL1A				
Genotype				
CC	14	9	6.83	0.03
CT	25	5		
TT	6	0		
Allele				
C	53	23	5.46	0.02
T	37	5		
IL1B				
Genotype				
CC	21	9	2.55	0.28
CT	21	5		
TT	3	0		
Allele				
C	63	23	1.67	0.19
T	27	5		

Distribution of the IL1A (-889) and IL1B (+3954) genotypes and allele in toxoplasmosis retinochoroiditis (TR) patients followed for one or more years who presented (Yes; n=35) or (No; n=14) further recurrence of the disease.

As for *IL1B* +3954 C/T polymorphism analysis, no difference was found between observed and expected distributions of genotypes for the control group and therefore it was considered to be in Hardy–Weinberg equilibrium. There was no significant difference in the genotype (χ^2^=3.12; p=0.21) or allele (χ^2^=1.92; p=0.17) distribution in patients with TR compared with control subjects (Table 1). The frequencies of the genotype (χ^2^=2.55; p=0.28) and allele (χ^2^=1.67; p=0.19) distributions did not significantly differ between TR patients with and without recurrent disease (Table 2).

## Discussion

In our study, we found that recurrence of TR is associated with *IL1A* −889 C/T polymorphism. The CT genotype and T allele were associated with TR recurrence. This link between TR and a genotype or allele associated with a high IL-1α production provides evidence that abnormalities in the genetic control of cytokine levels may be relevant in influencing the immune response in TR.

The inflammatory response in uveitis is controlled by a series of mediators including cytokines that seem to be locally produced in aqueous humor [16]. Cytokine production has been shown to be under genetic control. Polymorphisms in cytokine genes may influence the susceptibility to inflammatory diseases or their severity. In line with our results, a previous study found that −889 T *IL1A* allele, related with higher IL-1α production, was associated with the development of chronic iridocyclitis in juvenile rheumatoid arthritis patients [17]. However, Cimaz, et al. [18] found no association when they investigated the genetic contribution of *IL1B* +3954 C/T polymorphism on uveitis occurrence in juvenile rheumatoid arthritis patients.

There is evidence that *IL1A* and *IL1B* polymorphisms may be in linkage disequilibrium [19] and that the TT genotype of the *IL1A* −889 C/T polymorphism influences circulating levels of IL-1β [20]. However, in the present study, we did not find an association between *IL1B* +3954 C/T polymorphism and TR recurrence.

To date, few studies have analyzed the association of cytokine polymorphisms and TR. A previous study performed by us showed that the *IL10* −1082 A allele was associated with TR occurrence [21]. This allele is associated with intermediate or lower IL-10 production. Regarding IL-10 as a major anti-inflammatory cytokine, this result suggests that TR seemed to occur in patients who may have problems in controlling inflammatory responses. Interestingly, according to the present results, TR may recur in patients prone to develop a more intense inflammation. Thus, a high inflammatory profile may be linked with occurrence as well as recurrence of TR. Together these results provide evidence for the role of genetic control of cytokine levels in the pathogenesis of TR.

This study is the first to demonstrate the association between the genetic polymorphism and recurrence of TR in humans. Recurrence rates vary greatly. The confirmation of a role for IL-1α in TR will require larger cohorts of patients, longer follow-up, and the analysis of other polymorphisms or haplotypes.
